# Human platelet antigen 1-6, 9 and 15 in the Iranian population: An anthropological genetic analysis

**DOI:** 10.1038/s41598-020-64469-4

**Published:** 2020-05-04

**Authors:** Mohammad Hossein Kazemi, Farideh Malakootikhah, Zahra Momeni-Varposhti, Reza Falak, Ali-Akbar Delbandi, Nader Tajik

**Affiliations:** 1 Department of Immunology, School of Medicine, Iran University of Medical Sciences, Tehran, Iran; 2 Immunology research center, Iran University of Medical Sciences, Tehran, Iran; 3 Hematopoietic Stem Cell Research Center, Shahid Beheshti University of Medical Sciences, Tehran, Iran

**Keywords:** Classification and taxonomy, Structural variation, Coagulation system, Immunogenetics, Genetics research

## Abstract

Human platelet antigens (HPAs) are membranous glycoproteins considered as alloantigens due to their polymorphisms. HPA-incompatibility in multiple pregnancies or blood transfusion can induce the development of alloantibodies leading to thrombocytopenia. The frequency of HPAs varies among populations, so that deep knowledge of HPA frequencies will help us to reduce those incompatibilities. Herein, we studied the allele and genotype frequencies of HPA1-6, HPA9, and HPA15 among the Iranians with intra- and inter-populations analyses on 36 worldwide populations with diverse ethnicities. The analysis shows that the HPA2 and HPA5 have the greatest differences in genotype distribution between the Iranians and other nations, although similar to other populations, the sole allele found in HPA4, 6, and 9 is “a”. Despite other HPAs, the most frequent allele in HPA15 is “b”, which is also abundant in HPA3. Hierarchical clustering indicates the highest degree of global similarity in HPA genotype frequency among Iranian, Argentinian, Brazilian, and German Turkish populations. Our findings can be applied to decrease the risk of alloimmunizations and platelet disorders, especially in neonates.

## Introduction

Platelets are tiny, essential blood components that were previously considered only in homeostasis by adherence to damaged vessels, thus creating clots with fibrin to prevent bleeding. Nowadays, they have been assigned a much greater diversity of roles beyond clotting, such as in inflammation, innate and adaptive immunity^1,2^, proving these small objects to be critical in a wide range of diseases^1^. Most platelet functions are performed by binding to other cells through membranous glycoproteins (GPs). On these GPs, there are polymorphic amino acid sequences called human platelet antigens (HPAs), which are considered alloantigens due to their polymorphism^3^. So far, 41 types of HPA have been serologically identified, the naming of which is in the order of recognized identification^4^. Twelve antigens, including HPA-1a/1b, -2a/2b, -3a/3b, -4a/4b, -5a/b, and -15a/b are biallelic and located on GPIIIa, GPIba, GPIIb, GPIIIa, GPIa and CD109, respectively^5^. HPAs are inherited in a codominant autosomal way, and the most frequent allele is called “a”^6,7^. Their polymorphism is mainly because of the alteration of an amino acid within their structure, following a single nucleotide polymorphism (SNP). One amino acid alteration can cause a change in the tertiary structure of the antigen, leading to new epitope creation capable of inducing the alloantibodies production^3^.

Incompatibility in platelet antigens between mother and fetus during pregnancy or donor and recipient in case of blood transfusion can induce alloantibodies which may destroy platelets, thus creating thrombocytopenia and various hemorrhagic disorders, including fetal and neonatal alloimmune thrombocytopenia (FNAIT), post-transfusion purpura (PTP) and multi-transfusion platelet refractoriness (MPR)^5,8,9^. Thrombocytopenia is a common, fatal complication in hematological diseases, which proves the importance of HPAs in clinical researches more than before. The most immunogenic HPAs are HPA1a and HPA5b, which are responsible for inducing more than 90% of antiplatelet alloantibodies^10^.

The frequency of HPAs varies among populations, and thorough knowledge of HPA frequencies among different populations can play an important role in reducing antigen differences between donor and recipient, hence diminishing the risk of alloimmunization. In fact, determining HPA allele and genotype frequency among populations can provide a significant basis in reducing the problems associated with the HPA-mediated alloreactions in suspected or highly alloimmunized individuals.

This study represents the first report of HPA1-6, HPA9, and HPA15 allele and genotype frequency within the Iranian population, with a comparison of such frequencies to those reported in former studies.

## Results

### Intra-population study

The allele and genotype frequencies of HPA1-6, 9, and 15 in 300 healthy Iranian subjects are illustrated in Table 1. The distribution of the genotypes for all HPA genes does not show any significant deviation from the Hardy-Weinberg equilibrium (HWE), except for HPA5, which is different from HWE (p-value=0.011, X^2^ = 6.33). In contrast to other HPAs, the most frequent allele seen in HPA15 is “b” (54%), with the highest ratio of “ab” and “bb” genotype frequencies (49.3 and 29.4%, respectively). HPA3 has also a high proportion of allele “b” (41.3%), with a high frequency of “ab” and “bb” genotypes (43.3 and 19.7%, respectively). The greatest amount of “a” allele can be observed in HPA4, 6, and 9, which comprises all alleles, without any “b” allele. Accordingly, all the genotypes in HPA4, 6, and 9 are “aa”, and no “ab” or “bb” genotypes is detected. The only HPA having the allele “b”, but no homozygous “bb” genotype, is HPA1, in which all the allele “b” is in the heterozygous form.

**Table 1:** Allele and genotype distribution of HPA1-6, 9 and 15 in the Iranian population.

HPA	Alleles/genotypes	Healthy Control N (%)	P^a^
HPA1	a	541 (90.2)	Ref.
	b	59 (9.8)	**<0.001**
	aa	241(80.3)	Ref.
	ab	59 (19.7)	**<0.001**
	bb	0	NA
HWE		X^2^ = 3.57	0.058
HPA2	a	528 (88)	Ref.
	b	72 (12)	**<0.001**
	aa	232 (77.3)	Ref.
	ab	64 (21.3)	**<0.001**
	bb	4 (1.4)	<**0.001**
HWE		X^2^ = 0.03	0.862
HPA3	a	352 (58.7)	Ref.
	b	248 (41.3)	**<0.01**
	aa	111 (37)	Ref.
	ab	130 (43.3)	0.114
	bb	59 (19.7)	**<0.01**
HWE		X2 = 3.4	0.065
HPA4	a	600 (100)	Ref.
	b	0	NA
	aa	300 (100)	Ref.
	ab	0	NA
	bb	0	NA
HWE		X^2^ = NA	NA
HPA5	a	546 (91)	Ref.
	b	54 (9)	**<0.001**
	aa	252 (84)	Ref.
	ab	42 (14)	**<0.001**
	bb	6 (2)	<**0.001**
HWE		X^2^ = **6.33**	**0.011**
HPA6	a	600 (100)	Ref.
	b	0	NA
	aa	300 (100)	Ref.
	ab	0	NA
	bb	0	NA
HWE		X^2^ = NA	NA
HPA9	a	600 (100)	Ref.
	b	0	NA
	aa	300 (100)	Ref.
	ab	0	NA
	bb	0	NA
HWE		X^2^ = NA	NA
HPA15	a	276 (46)	**<0.01**
	b	324 (54)	Ref.
	aa	64 (21.3)	**<0.05**
	ab	148 (49.3)	<**0.001**
	bb	88 (29.4)	Ref.
HWE		X^2^ = 0.01	0.920

ªAdjusted p-value for multiple testing using Benjamini-Hochberg method; OR. odds ratio; HWE. Hardy-Weinberg equilibrium; NA. not available.

The most frequent genotypes and haplotypes among the population under study with a relative frequency of more than 1% of the population are listed in Table 2. The genotypes comprise all HPA studied (HPA1-6, 9, and 15), and the haplotypes consist of the HPAs that are located on a same chromosome (HPA1-4, 6, and 9 on the chromosome 17). There are 28 genotypes with a frequency ratio of over 1%, comprising more than 87% of the population, and the genotype “HPA1aa, HPA2aa, HPA3ab, HPA4aa, HPA5aa, HPA6aa, HPA9aa, HPA15ab” shows the highest frequency (9.33%). The first four genotypes include more than one-third of the whole population. Eleven frequent haplotypes cover 98.4% of the population, in which the most frequent haplotype is “HPA1aa, HPA2aa, HPA3ab, HPA4aa, HPA6aa, HPA9aa”, with a frequency rate of 29%, and the first two haplotypes cover more than half of the population.

**Table 2:** The most frequent genotypes and haplotypes of HPA1-6, 9 and 15 in the Iranian population.

Genotypes	N (%)	Haplotypes	N (%)
1aa2aa3ab4aa5aa6aa9aa15ab	28 (9.33)	1aa2aa3ab4aa6aa9aa	87 (29)
1aa2aa3aa4aa5aa6aa9aa15ab	25 (8.33)	1aa2aa3aa4aa6aa9aa	66 (22)
1aa2aa3ab4aa5aa6aa9aa15bb	25 (8.33)	1aa2aa3bb4aa6aa9aa	34 (11.3)
1aa2aa3ab4aa5aa6aa9aa15aa	19 (6.33)	1aa2ab3ab4aa6aa9aa	21 (7)
1aa2aa3aa4aa5aa6aa9aa15bb	17 (5.66)	1ab2aa3aa4aa6aa9aa	20 (6.7)
1aa2aa3bb4aa5aa6aa9aa15ab	14 (4.66)	1aa2ab3aa4aa6aa9aa	19 (6.3)
1ab2aa3aa4aa5aa6aa9aa15ab	14 (4.66)	1ab2aa3ab4aa6aa9aa	14 (4.7)
1aa2ab3ab4aa5aa6aa9aa15ab	11 (3.66)	1aa2ab3bb4aa6aa9aa	12 (4)
1aa2aa3aa4aa5aa6aa9aa15aa	10 (3.33)	1ab2aa3bb4aa6aa9aa	11 (3.7)
1aa2ab3aa4aa5aa6aa9aa15ab	10 (3.33)	1ab2ab3ab4aa6aa9aa	6 (2)
1aa2aa3ab4aa5ab6aa9aa15ab	8 (2.66)	1ab2ab3aa4aa6aa9aa	5 (1.7)
1aa2aa3bb4aa5aa6aa9aa15aa	8 (2.66)		
1aa2aa3bb4aa5aa6aa9aa15bb	7 (2.33)		
1ab2aa3ab4aa5aa6aa9aa15ab	7 (2.33)		
1aa2aa3aa4aa5ab6aa9aa15bb	5 (1.66)		
1aa2aa3aa4aa5ab6aa9aa15ab	5 (1.66)		
1aa2aa3ab4aa5ab6aa9aa15bb	5 (1.66)		
1aa2ab3aa4aa5aa6aa9aa15bb	5 (1.66)		
1aa2ab3bb4aa5aa6aa9aa15aa	5 (1.66)		
1ab2ab3ab4aa5aa6aa9aa15ab	5 (1.66)		
1aa2ab3ab4aa5aa6aa9aa15bb	4 (1.33)		
1aa2ab3bb4aa5aa6aa9aa15ab	4 (1.33)		
1ab2aa3bb4aa5aa6aa9aa15aa	4 (1.33)		
1ab2ab3aa4aa5aa6aa9aa15ab	4 (1.33)		
1ab2aa3ab4aa5aa6aa9aa15bb	4 (1.33)		
1aa2ab3ab4aa5aa6aa9aa15aa	3 (1)		
1aa2aa3bb4aa5ab6aa9aa15ab	3 (1)		
1ab2aa3bb4aa5aa6aa9aa15ab	3 (1)		

Genotypes and haplotypes with relative frequency more than 1% were selected for analysis.

The linkage disequilibrium coefficients were evaluated to examine the possible linkages between HPA genes in heritage, and no significant linkage disequilibrium was found between the investigated HPAs (data not shown).

### Inter-population study

Thirty-six populations were selected from previous works throughout the world, to compare the distribution of HPA genes between the Iranians and other nations. The comparison of genotype distribution between our study and other populations are analyzed and shown in Table 3. Among HPA1, 2, 3, 5, and 15, the distribution of HPA2 and HPA5 genotypes are significantly different between the Iranians and 23 populations. The results shows that almost 64% of the studied populations have different HPA2 and HPA5 genotype distribution, compared to the Iranians. The genotype distribution in HPA3, 1, and 15 is statistically different in roughly 60%, 55%, 48% of the populations, in comparison with those of the Iranians.

**Table 3:** Comparison of genotype distributions of HPA1-6, 9 and 15 between this study and other studies based on the populations.

Population	N	HPA1			HPA2			HPA3			HPA4			HPA5			HPA6			HPA9			HPA15			Ref.
		aa	ab	bb	aa	ab	bb	aa	ab	bb	aa	ab	bb	aa	ab	bb	aa	ab	bb	aa	ab	bb	aa	ab	bb	
Iranian (This study)	300	0.8	0.2	0	0.78	0.2	0.02	0.37	0.43	0.2	1	0	0	0.84	0.14	0.02	1	0	0	1	0	0	0.21	0.49	0.3	—
Moroccan Berber	**110**	**0.58**^**‡**^	0.34	0.08	**0.66**^**‡**^	0.3	0.04	**0.49**^**‡**^	0.38	0.13	1	0	0	0.73	0.26	0.01	1	0	0	—	—	—	—	—	—	^25^
Tunisian	**116**	**0.6**^**‡**^	0.37	0.03	0.74^*^	0.24	0.02	**0.66**^**‡**^	0.18	0.16	1	0	0	**0.64**^**‡**^	0.32	0.04	1	0	0	—	—	—	**0.28**^**‡**^	0.46	0.26	^35^
Beninese	154	0.8	0.19	0.01	**0.48**^**‡**^	0.45	0.07	**0.46**^**‡**^	0.43	0.11	1	0	0	**0.64**^**‡**^	0.35	0.01	1	0	0	1	0	0	**0.44**^**‡**^	0.41	0.15	^26^
Cameroonians	118	0.82	0.17	0.01	**0.6**^**‡**^	0.32	0.08	**0.36**^**‡**^	0.5	0.14	1	0	0	**0.57**^**‡**^	0.35	0.08	1	0	0	1	0	0	**0.5**^**‡**^	0.38	0.12	^26^
Pygmies	111	1	0	0	**0.35**^**‡**^	0.53	0.12	**0.24**^**‡**^	0.51	0.25	1	0	0	**0.37**^**‡**^	0.45	0.18	1	0	0	1	0	0	**0.51**^**‡**^	0.33	0.16	^26^
Egyptian	**367**	**0.62**^**‡**^	0.34	0.04	0.75	0.23	0.02	0.43^*^	0.45	0.12	1	0	0	**0.71**^**‡**^	0.27	0.02	—	—	—	—	—	—	0.28^*^	0.5	0.22	^27^
Congolese	125	0.82	0.17	0.01	**0.59**^**‡**^	0.37	0.04	0.36	0.47	0.17	1	0	0	**0.52**^**‡**^	0.42	0.06	1	0	0	1	0	0	**0.49**^**‡**^	0.42	0.09	^26^
Jordanian	**116**	**0.66**^**†**^	0.32	0.02	0.77	0.21	0.02	0.47^*^	0.38	0.15	1	0	0	**0.61**^**‡**^	0.37	0.02	—	—	—	—	—	—	—	—	—	^30^
Saudi	**100**	0.75^*^	0.23	0.02	**0.62**^**†**^	0.32	0.06	0.51^*^	0.39	0.1	0.99	0.01	0	0.76^*^	0.24	0	1	0	0	—	—	—	0.2	0.5	0.3	^29^
Bahraini	**194**	**0.57**^**‡**^	0.38	0.05	**0.6**^**‡**^	0.33	0.07	0.3	0.51	0.19	0.87	0.12	0.01	0.76	0.21	0.03	—	—	—	—	—	—	—	—	—	^28^
Indonesian	500	0.95	0.05	0	**0.88**^**‡**^	0.12	0	**0.27**^**†**^	0.48	0.25	0.89	0.1	0.01	**0.95**^**‡**^	0.04	0.01	0.91	0.09	0	—	—	—	**0.26**^**‡**^	0.58	0.16	^18^
Malay	**200**	**0.95**^**‡**^	0.05	0	**0.92**^**‡**^	0.08	0	0.24^*^	0.51	0.25	0.99	0	0.01	0.9^*^	0.1	0	0.98	0.02	0	—	—	—	0.25	0.53	0.22	^19^
Taiwanese	998	0.99	0.01	0	**0.93**^**‡**^	0.06	0.01	0.3^*^	0.52	0.18	0.99	0.01	0	**0.97**^**‡**^	0.03	0	0.95	0.04	0.01	—	—	—	**0.28**^**†**^	0.51	0.21	^20^
Chinese	1000	0.99	0.01	0	**0.9**^**‡**^	0.09	0.01	0.35	0.47	0.18	0.99	0.01	0	**0.97**^**‡**^	0.02	0.01	0.97	0.03	0	1	0	0	**0.28**^**†**^	0.5	0.22	^53^
Vietnamese	107	0.97	0.03	0	**0.9**^**†**^	0.1	0	0.22^*^	0.54	0.24	1	0	0	0.94^*^	0.06	0	0.97	0.03	0	1	0	0	0.24	0.57	0.19	^54^
Indian	**1164**	**0.87**^**‡**^	0.12	0.01	**0.99**^**‡**^	0.01	0	**0.01**^**‡**^	0	0.99	0.99	0.01	0	**0.79**^**‡**^	0	0.21	0.99	0	0.01	—	—	—	—	—	—	^36^
Thai	500	0.97	0.03	0	**0.9**^**‡**^	0.1	0	0.3	0.51	0.19	1	0	0	**0.93**^**‡**^	0.07	0	0.97	0.03	0	—	—	—	0.25	0.48	0.27	^15^
Burmese	285	0.89	0.11	0	**0.94**^**‡**^	0.06	0	**0.35**^**‡**^	0.5	0.15	0.99	0.01	0	**0.97**^**‡**^	0.03	0	0.97	0.03	0	—	—	—	**0.33**^**‡**^	0.28	0.39	^16^
Karen	242	0.95	0.05	0	**0.92**^**‡**^	0.08	0	0.3	0.5	0.2	0.99	0.01	0	**0.98**^**‡**^	0.02	0	0.98	0.2	0	—	—	—	0.27	0.51	0.22	^16^
North-eastern Thais	**300**	**0.94**^**‡**^	0.06	0	**0.88**^**‡**^	0.11	0.01	0.38^*^	0.49	0.13	1	0	0	**0.92**^**‡**^	0.08	0	0.97	0.03	0	—	—	—	0.24	0.52	0.24	^16^
Koreans	**200**	**0.98**^**‡**^	0.02	0	0.85	0.13	0.02	**0.28**^**‡**^	0.54	0.18	0.98	0.02	0	**0.95**^**‡**^	0.05	0	0.96	0.04	0	—	—	—	—	—	—	^17^
Japanese	100	1	0	0	0.78	0.2	0.02	0.43	0.4	0.17	1	0	0	0.93	0.06	0.01	0.98	0.02	0	—	—	—	—	—	—	^32^
Pakistani	593	0.8	0.19	0.01	0.85^*^	0.13	0.02	**0.51**^**‡**^	0.36	0.13	1	0	0	0.82	0.16	0.02	—	—	—	—	—	—	—	—	—	^31^
Germany Caucasian	**119**	**0.63**^**‡**^	0.33	0.04	0.81	0.19	0	0.33	0.47	0.2	1	0	0	0.85	0.13	0.02	—	—	—	—	—	—	0.27	0.49	0.24	^23^
Germany Turkish	**117**	**0.76**^**†**^	0.2	0.04	0.76	0.21	0.03	0.42	0.37	0.21	0.99	0.01	0	0.79	0.2	0.01	—	—	—	—	—	—	0.2	0.54	0.26	^23^
Norway	**105**	**0.77**^**†**^	0.2	0.03	0.89^*^	0.1	0.01	0.24^*^	0.44	0.32	1	0	0	0.86	0.12	0.02	—	—	—	—	—	—	0.27	0.44	0.29	^24^
Wales	**392**	**0.68**^**‡**^	0.28	0.04	0.8	0.2	0	0.38	0.46	0.16	1	0	0	0.81	0.18	0.01	1	0	0	—	—	—	—	—	—	^22^
Danish	**557**	**0.7**^**‡**^	0.26	0.04	0.84^*^	0.15	0.01	**0.37**^**†**^	0.51	0.12	1	0	0	**0.92**^**†**^	0.08	0	—	—	—	—	—	—	—	—	—	^21^
Canadian	**750**	**0.72**^**†**^	0.26	0.02	**0.86**^**†**^	0.13	0.01	**0.43**^**†**^	0.45	0.12	1	0	0	0.8	0.19	0.01	—	—	—	—	—	—	0.27^*^	0.51	0.22	^33^
White American	**100**	0.8^*^	0.18	0.02	0.85	0.12	0.03	0.46	0.42	0.12	1	0	0	0.79	0.19	0.02	—	—	—	—	—	—	—	—	—	^34^
Black American	100	0.84	0.16	0	0.67	0.3	0.03	0.4	0.45	0.15	1	0	0	**0.62**^**‡**^	0.34	0.04	—	—	—	—	—	—	—	—	—	^34^
Australian Caucasians	**1000**	**0.73**^**†**^	0.25	0.02	**0.85**^**‡**^	0.14	0.01	0.38^*^	0.48	0.14	1	0	0	0.82	0.17	0.01	—	—	—	—	—	—	—	—	—	^55^
Australian aboriginals	185	0.99	0.01	0	1	0	0	**0.87**^**‡**^	0.11	0.02	1	0	0	**0.56**^**‡**^	0.38	0.06	—	—	—	—	—	—	—	—	—	^55^
Ma’ohis Polynesian	**81**	**0.95**^**‡**^	0.05	0	0.83	0.17	0	0.43	0.37	0.2	1	0	0	0.95^*^	0.05	0	0.83	0.17	0	1	0	0	0.28	0.52	0.2	^54^
Argentinian	192	0.76	0.22	0.02	0.77	0.2	0.03	0.4	0.43	0.17	1	0	0	0.86	0.12	0.02	1	0	0	—	—	—	0.24	0.53	0.23	^56^
Brazilian	**158**	**0.74**^**†**^	0.22	0.04	0.77	0.2	0.03	0.43	0.44	0.13	0.99	0.01	0	0.83	0.15	0.02	1	0	0	0.99	0.01	0	0.3	0.46	0.24	^37^

Genotype frequencies of HPA1-6, 9 and 15 in the populations are shown. Bold values indicate the statistical significant differences between HPA genotype frequency with those of the Iranian population. *P-value <0.05, ^†^P-value<0.01, ^‡^P-value < 0.001.

**Figure 1:**
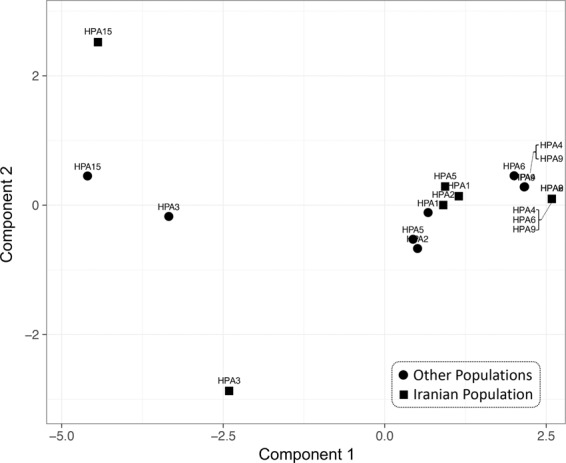
Principal component analysis (PCA) based on HPA gene alleles in the Iranian population and other studied populations. The pattern of HPA alleles in the Iranian population is shown as black squares, while the pattern of those in other studied populations is integrated and illustrated as black circles.

The two-dimensional principal component analysis was performed to illustrate the comparison in the HPA allele distribution pattern between the present and previous studies across other populations (Fig. 1). The overall pattern of HPA distribution in 36 populations is integrated and demonstrated as “the world HPA distribution pattern”, along with the HPA distribution pattern in the Iranian population. The frequency distribution of HPA1, 2, and 5 are close together, while HPA4, 6, and 9 is shown to be identical and their spots are completely overlapping. Similarly, the world distribution patterns of HPA1, 2, and 5 are close together, and the pattern of HPA4, 6, and 9 distributions is similar to the Iranian population, with the overlapping spots of HPA4 and 9. HPA3 and HPA15 are located far away, compared to other HPAs in both Iranian and world population patterns. However, these two HPAs are also located far from each other in the Iranian population pattern.

**Figure 2:**
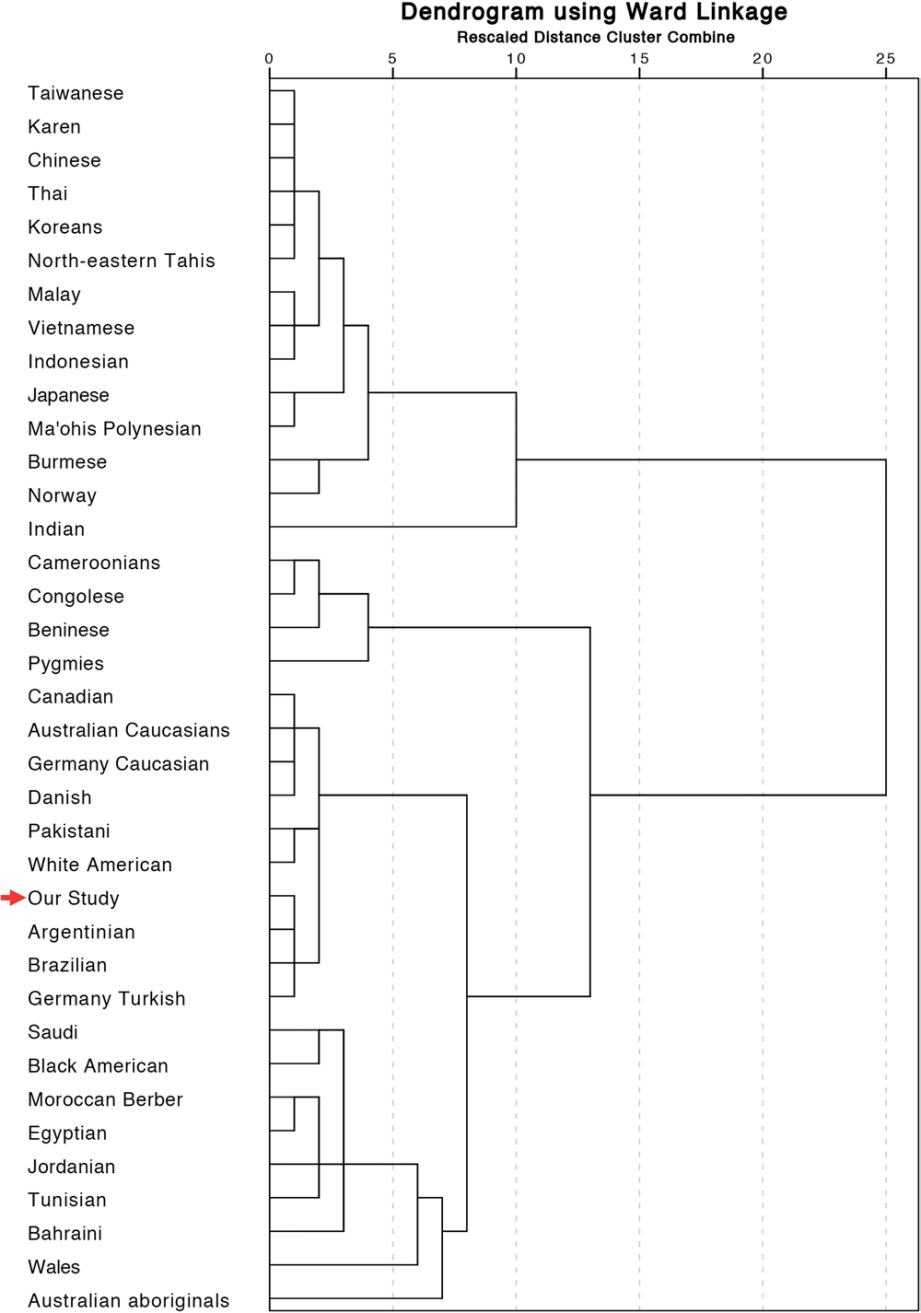
Hierarchical clustering of populations based on similarity of HPA genotype distribution. The populations are clustered, based on Ward Linkage and our study is highlighted with a red arrow.

Hierarchical population clustering based on the genotype distribution similarity of the HPAs under study, is illustrated in Fig. 2. It shows that the Iranian population is clustered with the Argentinian, Brazilian, and German Turkish populations at the first level. The populations, which are grouped with our study at the second level, are White American, Pakistani, Danish, German Caucasian, Australian Caucasian, and Canadian. Further details are represented in Fig. 2.

## Discussion

In addition to platelets, HPAs are expressed on other cells such as endothelial cells and monocytes, which is reported to probably behave as histocompatibility antigens in organ transplantation^11,12^. Although the mere HPA incompatibility between donor and recipient cannot lead to graft rejection, the possible alloimmunization might aggravate the transplant complications^13^. HPA genotyping could serve as a helpful tool in decreasing the consequences of alloimmune platelet disorders such as FNAIT. Many HPAs seem to have the capacity to induce maternal alloantibodies causing FNAIT, of which HPA1a and HPA5b account for about 90% of antiplatelet alloantibodies^10,14^.

This work represents the first comprehensive study on the distribution of human platelet antigen genotypes and alleles in the Iranian population with intra- and inter-populations analyses of 36 worldwide populations of diverse ethnicities.

The first revealing and unexpected finding is that the HPA5 frequency distribution does not follow the HWE. Regarding the fact that the individuals involved in this research originate from all over the country, with various ethnicities, the deviation from HWE might be due to the relatively small sample size of the study. More comprehensive investigations are very likely to determine the reason for such deviation. However, the accordance of other HPAs distributions with HWE might prove the validity of sample selection. The only HPA with “b” allele but no “bb” genotype is HPA1, indicating that all “b” alleles are in the heterozygous form. This pattern can also be seen in most East-Asian countries^15–20^, though not common among Europeans^21–24^, Africans^25–27^, and other people in the Middle-East^28–31^. It is noteworthy to mention that, among the 36 populations studied, the Pygmies and Japanese showed merely “a” allele and “aa” genotype in HPA1^26,32^. Regarding that no HPA1bb genotype is found in our study and all individuals have “a” allele in HPA1, at least in heterozygous form, it could be concluded that, in the Iranian population, antibodies against HPA1a are probably not as important as those nations, such as North Americans^33,34^, North Europeans^2,21,23,24^, and some Africans^25–27,35^, in which the homozygous form (HPA1bb) is reported.

Most differences in the HPA genotype distribution between the Iranians and other nations can be seen in HPA2 and HPA5, among which 64% of the populations have significantly different genotype patterns compared to the Iranians. Given the deviation of HPA5 from HWE, such a result is difficult to interpret. Concerning HPA2, this difference proves to be significant and should, therefore, be taken into account. The HPA3 shows a high-frequency ratio in allele “b”, with the frequent occurrence of both “ab” and “bb” genotypes observed in other populations, except for the Indians, for whom, interestingly, the “b” homozygosity has been detected in almost all studied individuals, with only one “aa” genotype and no “ab” genotype has been presented^36^. However, the HPA3 genotype distribution in our study is also significantly different from 60% of the studied populations. According to many reports, the sole allele found in HPA4, 6, and 9 is “a”, thus leading to the “aa” genotype in all participants in this study. However, in some nations, the HPA4 and 6 heterozygote forms have been reported, as well as the homozygote form of “bb” for HPA4 in Bahraini, Indonesian, and Malay populations^18,19,28^, and concerning HPA6, in Taiwanese people and Indians^20,36^.

Interestingly, there is no report of “bb” genotype, and only one report of “ab” genotype for HPA9 (Brazilians) was found among the 36 populations mentioned^37^. Contrary to other HPAs, in which the most frequent allele found is “a”, in the case of HPA15, the most common allele is “b” and, accordingly, the highest frequency of “ab” and “bb” genotypes appears in the latter HPA. The “bb” genotype rates in HPA15 in all the populations under study are proved to be lower than those of the Iranians, except for Burmese population^16^, with 39%, and the Saudi Arabs^29^, with 30% of the homozygous genotype of allele “b”. Inversely, the “aa” genotype frequency in HPA15 in the Iranian population is lower than the other nations. However, those of Saudi Arabia (20%) and German Turkish (20%) populations show an “aa” frequency of only 1% lower than the Iranians^23,29^. This might be indicative of a slight drift from allele “a” to allele “b” in HPA15 among the Iranian population.

As demonstrated in Table 2, there are 28 frequent HPA genotypes that comprise more than 87% of the people. The remaining 13% have rare genotypes, which should be taken into account in the case of alloimmunization. The most frequent genotype is “HPA1aa, HPA2aa, HPA3ab, HPA4aa, HPA5aa, HPA6aa, HPA9aa, HPA15ab”, observed in 9.33% of the individuals. One-third of the whole population demonstrates one of the four most frequent genotypes. To evaluate the HPA haplotypes distribution within the population, the frequencies of HPA1-4, 6, and 9 that are encoded by genes in a same chromosome (chromosome 17) was studied. More than 98% of the population is classified into eleven frequent HPA haplotypes, in which the most common haplotype is “HPA1aa, HPA2aa, HPA3ab, HPA4aa, HPA6aa, HPA9aa”, with a frequency rate of 29%, and the first two haplotypes involve more than half of the population. As expected, the most recurrent haplotype is proved to be in accordance with the most frequent genotype. Hence, it could be concluded that the disparities in HPA5 and 15 account for 20% difference between the frequency of the major genotype and haplotype.

The linkages between the HPA genes in heritage were also examined through the use of linkage disequilibrium coefficients, although no significant linkage disequilibrium was found between the HPAs under study. Such a finding suggests that, unlike HLA, the heritage of HPA genes might be independent of each other^38^. However, linkage disequilibrium between some HPAs has been reported in some studies^39^.

According to the two-dimensional principal component analysis illustrated in Fig. 1, the HPA allele overall distribution pattern in the present study is similar to those of previous works across other populations. In both the Iranians and integrated world population, the frequency distribution of HPA1, 2, and 5 are close together, thereby indicating that the frequencies of “a” and “b” alleles in these three HPAs are comparable. Despite the deviation of the HPA5 frequency distribution from HWE, the comparable pattern of allele distribution in HPA5 between the Iranians and the world could validate the sample selection of the present study. On the other hand, the predominance of allele “a” in HPA4, 6, and 9 in both populations render their spots as neighbors. HPA3 and HPA15 are located far away in comparison with the other HPAs in both Iranian and world population patterns. However, these two HPAs are also distant from each other in the Iranian population pattern, whereas relatively close in the integrated world population. Such a result suggests that the frequency pattern in the latter two HPAs among the Iranians presents some difference degree with the world population, deserving, as such, closer attention.

In order to determine the overall similarities between the studied populations with the aspect of HPA frequency, a hierarchical population clustering was undertaken based on the similarity of genotype distribution (Fig. 2). The clustering of the Iranians with the Argentinian, Brazilian, and German Turkish populations at the first level reveals the overall likeness between the frequencies of HPA genotypes in the abovementioned nations. The populations, which resemble our study at the second level, are White American, Pakistani, Danish, German Caucasian, Australian Caucasian, and Canadian populations. The resemblance of the Iranians with Caucasians might be explained by the fact that the northern parts of Iran are Caucasian. Interestingly enough, despite geographical proximity, the Middle East Arabs are located in another cluster, which proves the race differences between Iranians and Arabs. The similarity of HPA distribution among Middle Eastern Arabs and North Africans, and their contrasts with Eastern Asians have already been reported^30,40^. As could be expected, the Eastern Asian populations are clustered together, far from the Iranians, thus reflecting the genetic differences between the two areas.

One of the suggestions for future works is an inter-ethnic study within the Iranian population. Although we covered all ethnicities in sampling and made inter-ethnic comparisons, the results revealed no significant differences (data not shown), which might be due to the small sample size in each ethnicity. A reliable comparative study among different ethnicities of Iran requires larger sample sizes in further investigations. As an example, Brouk *et al*. reported HPA frequencies in 485 Algerian individuals from different ethnic groups^41^. We excluded this study from our analyses due to the lack of a united report from Algeria; however, there might be some differences, especially in the frequency of HPA3 between Iranians and Algerian ethnic groups.

Another interesting era, which is strongly suggested to be investigated, is the frequency of the Iranians who are deficient in expressing GPIV/CD36, another important platelet alloantigen. CD36 deficiency, which is observed in 2–8% of Asian and African people^42–45^, can cause alloimmunization in pregnancy and transfusions. A Chinese study on 53 Iranian blood donors reported 1.89% CD36 deficiency that needs larger confirmatory investigations^45,46^.

In conclusion, we attempted to characterize polymorphisms in 8 HPA systems among the Iranian population and comparing them with those of the datasets selected from previous studies on worldwide populations, in order to determine any discrepancies between them. We found greatest differences in HPA2 and HPA5 genotype distribution between the Iranians and other nations. Despite other HPAs, the most frequent allele in HPA15 is “b”, which is also abundant in HPA3. We also found the highest degree of global similarity in HPA genotype frequency between Iranians, Argentinian, Brazilian, and German Turkish populations in hierarchical clustering. Such comprehensive analyses of HPA polymorphisms in various populations can be applied as a basis of future investigations to decrease the risk of alloimmunizations^29,47–49^.

## Methods

### Subjects and genomic DNA extraction

The present study was conducted with 300 healthy unrelated Iranian individuals from all ethnicities in order to be generalized to the entire Iranian population. The subjects consisted of 179 men and 121 women with a mean age of 59.9 ± 9.9, presenting no autoimmune disorders, malignancies, or other underlying diseases. All participants signed informed consent, and the research project received ethics approval by the Ethics Committee of Iran University of Medical Sciences, Tehran, Iran (IR.IUMS.FMD.REC.91.17657). All procedures and methods were carried out in accordance with the relevant guidelines and regulations, as cited in the related study in each section. 5 mL venous blood was collected from each subject in ethylene diamine tetraacetic acid (EDTA) tube, and whole genomic DNA was isolated using the salting-out method^50^. The quantity and quality of extracted DNAs were determined by ultraviolet spectrophotometry.

### HPA polymorphism genotyping

**Figure 3:**
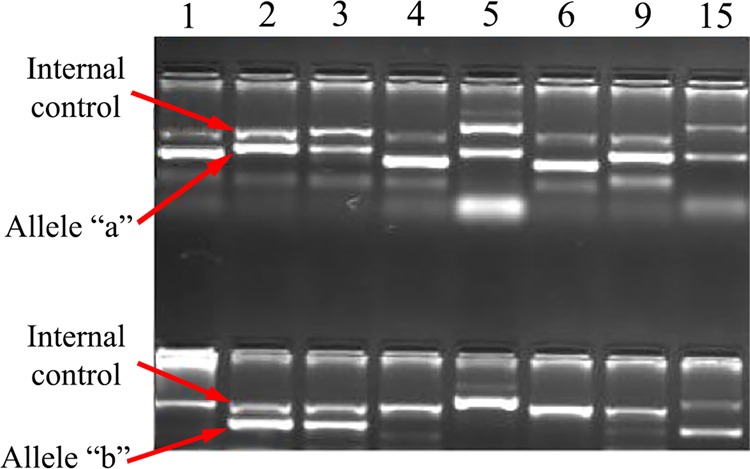
PCR-SSP genotyping electrophoresis. Full-length not-manipulated agarose gel electrophoresis of the PCR-SSP products. Representative typing results revealing the identification of homozygous a/a (HPA 1,4, 5, 6, and 9) heterozygous a/b (HPA 2,3, and 15). The upper arrow indicates a 429 bp of human growth hormone gene as internal amplification control for HPA 1, 2,3, 4, 6, 9 and a 607 bp of DRα gene as internal control for HPA 5 and 15, while the lower arrow indicates specific HPA allele.

The genotyping of 8 HPA polymorphisms (HPA1, 2, 3, 4, 5, 6, 9, and 15) was determined by polymerase chain reaction with the sequence-specific primers (PCR–SSP) method, previously described by Gaudet *et al*.^51^. Two sets of primers, each containing an allele-specific and a ‘common’ primer, were used for the detection of each allele^52^. A pair of human growth hormone (HGH) gene-specific primers at 429 bp was used as an internal control of HPA1-4, 6, 9 and a pair of DRα gene-specific primers at 607 bp was employed as an internal control of HPA5 and 15. The sequences of primers are shown in Table 4. Amplified products were detected by electrophoresis on (2% w/v) agarose gel stained with DNA safe stain. A representative HPA genotyping run is demonstrated in Fig. 3.

**Table 4:** Primers for HPA genotyping, using PCR-SSP.

Primer	Sequence	Tm (°C)	Amplicon Size
HPA-1a	5′ACTTACAGGCCCTGCCTCT 3′	62	196 bp
HPA-1b	5′ACTTACAGGCCCTGCCTCC 3′	62	
Common	5′AGCCGGAGTGCAATCCTCTG 3′	66	
HPA-2a	5′CCCCCAGGGCTCCTGAC 3′	64	241 bp
HPA-2b	5′GCCCCCAGGGCTCCTGAT 3′	62	
Common	5′GCCAGCGACGAAAATAGAGG 3′	62	
HPA-3a	5′GGGGGAGGGGCTGGGGA 3′	64	
HPA-3b	5′GGGGGAGGGGCTGGGGC 3′	66	
Common	5′GACCTGCTCTACATCCTGGA 3′	60	230 bp
HPA-4a	5′GCTGGCCACCCAGATGCG 3′	62	158 bp
HPA-4b	5′AGCTGGCCACCCAGATGCA 3′	60	
Common	5′GCTGTCCTGGCGTCTGGAG 3′	62	
HPA-5a	5′AGTCTACCTGTTTACTATCAAAG 3′	62	249 bp
HPA-5b	5′AGTCTACCTGTTTACTATCAAAA 3′	60	
Common	5′CTCTCATGGAAAATGGCAGTA 3′	62	
HPA-6a	5′GACGAGTGCAGCCCCCG 3′	60	163 bp
HPA-6b	5′GGACGAGTGCAGCCCCCA 3′	62	
Common	5′TAGCGGACACAGGAGAAGTC 3′	62	
HPA-9a	5′GGGCAGCCCCCAGTCCAC 3′	64	212 bp
HPA-9b	5′GGGCAGCCCCCAGTCCAT 3′	62	
Common	5′GACCTGCTCTACATCCTGGA 3′	62	
HPA-15a	5′TTCAAATTCTTGGTAAATCCTGT 3′	60	225 bp
HPA-15-b	5′TTCAAATTCTTGGTAAATCCTGG 3′	62	
Common	5′ATGACCTTATGATGACCTATTC 3′	60	
HGH-F	5′GCCTTCCCAACCATTCCCTTA 3′	64	429 bp
HGH-R	5′TCACGGATTTCTGTTGTGTTTC 3′	62	
DRα-F	5′GAGGTAACTGTGCTCACGAACAGC 3′	74	607 bp
DRα-R	5′CACGTTCTCTGTAGTCTCTGGG 3′	68	

HPA. Human platelet antigen; HGH. Human growth hormone; F. Forward; R. Reverse; Tm. Melting temperature; bp. base pair.

### Statistical methods

The allelic and genotypic associations of the HPA1-6, 9, and 15 were evaluated via Pearson’s χ2 test, using the SNP & Variation Suite software version 8.6.0 (Golden Helix, Bozeman, MT, USA). In addition, a comparison of HPA genotype distributions between Iran and other populations was conducted through the chi-square or Fisher exact test. The HPA genotype distributions were tested for deviation from Hardy-Weinberg equilibrium. P-values were corrected for multiple comparisons based on the Benjamini-Hochberg method. The pairwise linkage disequilibrium was calculated utilizing the Haploview version 4.2 software (Broad Institute, Cambridge, MA, USA). The hierarchical clustering between population was performed, based on the Ward linkage method by SPSS version 25 (SPSS Inc., Chicago, IL, USA).

## Data Availability

The dataset generated during this study is available from the corresponding author upon reasonable request.
